# Disparities in indications and outcomes reporting for pediatric tethered cord surgery: The need for a standardized outcome assessment tool

**DOI:** 10.1007/s00381-023-06246-y

**Published:** 2023-12-11

**Authors:** Matthew C. Findlay, Samuel Tenhoeve, Skyler A. Terry, Rajiv R. Iyer, Douglas L. Brockmeyer, Michael P. Kelly, John R. W. Kestle, David Gonda, Vijay M. Ravindra

**Affiliations:** 1 School of Medicine, University of Utah, Salt Lake City, UT USA; 2 Department of Neurosurgery, Clinical Neurosciences Center, University of Utah, Salt Lake City, UT USA; 3 College of Social and Behavioral Sciences, University of Utah, Salt Lake City, UT USA; 4 Division of Pediatric Orthopedics, Rady Children’s Hospital, San Diego, CA USA; 5 Division of Pediatric Neurosurgery, Rady Children’s Hospital, San Diego, CA USA; 6 Department of Neurological Surgery, Naval Medical Center San Diego, 34800 Bob Wilson Drive, San Diego, CA 92134 USA

**Keywords:** Tethered cord syndrome, Detethering, Pediatrics, Systematic review

## Abstract

**Purpose:**

Tethered cord syndrome (TCS) is characterized by abnormal attachment of the spinal cord neural elements to surrounding tissues. The most common symptoms include pain, motor or sensory dysfunction, and urologic deficits. Although TCS is common in children, there is a significant heterogeneity in outcomes reporting. We systematically reviewed surgical indications and postoperative outcomes to assess the need for a grading/classification system.

**Methods:**

PubMed and EMBASE searches identified pediatric TCS literature published between 1950 and 2023. Studies reporting surgical interventions, ≥ 6-month follow-up, and ≥ 5 patients were included.

**Results:**

Fifty-five studies representing 3798 patients were included. The most commonly reported non-urologic symptoms were nonspecific lower-extremity motor disturbances (36.4% of studies), lower-extremity/back pain (32.7%), nonspecific lower-extremity sensory disturbances (29.1%), gait abnormalities (29.1%), and nonspecific bowel dysfunction/fecal incontinence (25.5%). Urologic symptoms were most commonly reported as nonspecific complaints (40.0%). After detethering surgery, retethering was the most widely reported non-urologic outcome (40.0%), followed by other nonspecific findings: motor deficits (32.7%), lower-extremity/back/perianal pain (18.2%), gait/ambulation function (18.2%), sensory deficits (12.7%), and bowel deficits/fecal incontinence (12.7%). Commonly reported urologic outcomes included nonspecific bladder/urinary deficits (27.3%), bladder capacity (20.0%), bladder compliance (18.2%), urinary incontinence/enuresis/neurogenic bladder (18.2%), and nonspecific urodynamics/urodynamics score change (16.4%).

**Conclusion:**

TCS surgical literature is highly variable regarding surgical indications and reporting of postsurgical outcomes. The lack of common data elements and consistent quantitative measures inhibits higher-level analysis. The development and validation of a standardized outcomes measurement tool—ideally encompassing both patient-reported outcome and objective measures—would significantly benefit future TCS research and surgical management.

## Introduction

Tethered cord syndrome (TCS) is a neurosurgical condition characterized by an abnormal attachment of the spinal cord or neural structures to surrounding tissues, restricting its natural movement within the spinal canal. The pathophysiologic mechanism is thought to be stretching of the distal spinal cord with continued growth of the spinal column through childhood, resulting in spinal cord ischemia, diminished glucose use, a shift from oxidative to anaerobic metabolism, and metabolic failure [1–3]. TCS can be congenital or acquired and arises from various causes, including spinal cord injuries or postsurgical complications, leading to tension and stress on the caudal spinal cord and nerve roots [4]. As the neural elements “tether,” patients often experience symptoms including localized back pain, lower-extremity weakness, and bladder/bowel dysfunctions [4]. Other signs include foot clubbing, toe walking, and scoliosis [5, 6]. Prompt diagnosis and treatment are critical to alleviating symptoms and preventing further neurological impairment [7]. Because TCS commonly arises in utero and is rarely asymptomatic, it is most often diagnosed in pediatric populations, with an incidence of 0.25 per 1000 births [8, 9]. Managing adult TCS can be more nuanced, but surgery is almost always indicated in children [8–10]. With an aim of restoring cord mobility, tethered cord release (TCR) surgery can improve patient symptoms and quality of life [7].

Significant effort has since been spent to understand the natural history of and develop surgical techniques to optimize treatment for this condition [11]. Although the urologic, motor, and sensory symptoms associated with TCS have been well described [12], a standardized format or validated classification system to characterize presenting symptom severity and, more importantly, a measurement tool to track and compare postoperative outcomes are lacking. The absence of common data elements significantly inhibits the feasibility of nuanced meta-analyses and large-scale studies to inform surgical management. As novel surgical treatments for TCS, including spinal column shortening [13], become more commonplace, the need for a common language to compare outcomes is necessary. 

To characterize the disparity of differences in surgical indications and postsurgical outcomes for TCR, including column-shortening surgery, we performed a systematic review to identify which metrics are most commonly reported. We hypothesized that significant heterogeneity exists among surgical indications and outcomes reported for TCS and, ultimately, TCR. 

## Methods

### Information sources and search method

Studies were identified in August 2023 through a search of PubMed and EMBASE bibliographic databases for TCS detethering and column-shortening literature published from January 1950 through August 2023. The PubMed/Medline detethering searches comprised the MeSH primary term “tethered cord syndrome,” with secondary terms including “lipoma OR pediatric OR surgery OR child OR syndrome OR tethered cord OR untethering OR tethered spinal cord syndrome.” The EMBASE search included the terms “tethered cord syndrome,” “pediatrics,” and “surgery.” Cross-referencing was performed to ensure all potentially eligible studies were assessed. To capture any pediatric column-shortening TCS release studies, an additional search was performed in PubMed including “tethered cord shortening” OR “column shortening” OR “cord release”) AND (“tethered cord syndrome” OR “cord tethering”) AND (“surgery” OR “operative”) AND (“pediatrics” OR “children”). A similar EMBASE search included the terms “column shortening,” “pediatric,” “children,” and “tethered cord syndrome.” 

### Inclusion/exclusion criteria and study type

**Fig. 1:**
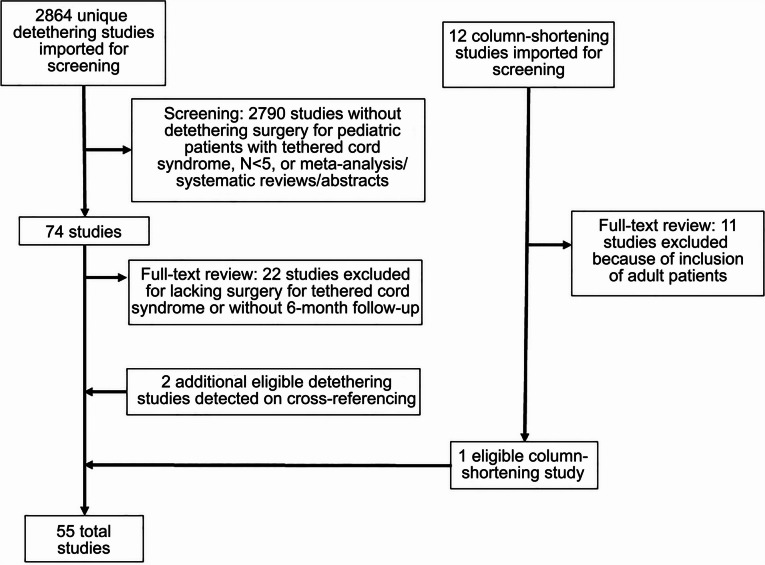
PRISMA inclusion/exclusion flowchart

Prospective and retrospective cohort studies and case series were reviewed. Reports with adult patients (> 18 years) or in which no patients underwent TCR or column shortening were removed. Additionally, review articles without new patient presentation, meta-analyses, systematic reviews, conference abstracts, and letters to the editor were excluded. Case reports and case series with < 5 patients were excluded to better identify common surgical indications and postoperative outcome variables and thereby improve the generalizability of this work. Studies lacking a 6-month follow-up were also removed. PRISMA guidelines [14] were followed in assembling this review (Fig. 1).

### Data collection

Relevant data were extracted by two independent reviewers (S.T. and S.A.T.). Any disagreements between reviewers were resolved by consensus and in consultation with M.F. If no consensus was reached, the disagreement was resolved by a pediatric neurosurgeon (V.M.R.). 

### Variables assessed

Each study was manually reviewed for the following categories, which were defined a priori: TCS etiologies, surgical indications, postoperative outcomes, and complications. The variables reported within each study were captured as encountered. Similar variables were coalesced into umbrella categories to increase readability (e.g., dermoid cyst and epidermoid cyst). When study variables were ill-defined, amorphous, or highly subjective, we reported these as “unspecified,” “general,” or “nonspecific.” Complications were captured as defined per the parameters of each study. Most notably, the characterization of secondary cord “retethering” as a complication event was not universal.

## Results

### Literature search

The database searches found 2876 unique studies for screening. Study quality and relevance were assessed using the Rayyan systematic review software (Cambridge, Massachusetts) [15]. After preliminary review, 86 studies remained for full-text analysis (Fig. 1). An additional 33 reports were then excluded. Cross-referencing revealed two additional studies eligible for inclusion, yielding a final cohort of 55 studies representing 3798 patients [10, 16–69]. In 54 studies, patients were managed with TCR (3778 patients), while the remaining study [68]. In 54 studies, patients were managed with TCR (3778 patients), while the remaining study [68] used spinal column shortening for “refractory” tethered cord with previous TCR (20 patients).

### Etiology of tethered cord

In total, we identified 38 anatomical defect categories (Table 1). The five most commonly reported were lipoma (reported by 60.0% of studies), thickened/fatty/tight filum terminale (43.6%), myelomeningocele (30.9%), lipomyelomeningocele (25.5%), and dermoid/epidermoid cysts (23.6%). Seven additional anatomical defect categories were reported in between 5 and 10 studies; 27 were used in ≤ 5 (9.1%) of the studies; and 11 defect types were reported by just a single study.

**Table 1:** Etiology of tethered cord

**Variable**	**Included studies (*n*** **= 55)**
Lipoma	33 (60.0)
Thickened/fatty/tight filum terminale	24 (43.6)
Myelomeningocele	17 (30.9)
Lipomyelomeningocele	14 (25.5)
Dermal sinus tract/dermoid cyst/epidermoid cyst	13 (23.6)
Syringomyelia/syrinx	9 (16.4)
Diastematomyelia	8 (14.5)
Caudal/sacral dysgenesis/agenesis	8 (14.5)
Meningocele	7 (12.7)
Unspecified spinal dysraphism	7 (12.7)
Low-lying conus medullaris	8 (14.5)
Lipomeningocele	6 (10.9)
Spina bifida occulta	5 (9.1)
Secondary tethered cord	5 (9.1)
Scoliosis	4 (7.3)
Split cord	4 (7.3)
Lipomyelocele	3 (5.5)
Arachnoid cyst	3 (5.5)
Unspecified spinal tumor	3 (5.5)
Myelocystocele	3 (5.5)
Unspecified spina bifida	2 (3.6)
Arnold-Chiari malformation	2 (3.6)
Neuroenteric cyst	2 (3.6)
Gluteal cleft deviation	2 (3.6)
Hemangioma	2 (3.6)
Teratoma	2 (3.6)
Chiari malformation type 1	1 (1.8)
Chiari malformation type 2	1 (1.8)
Meningocele manqué	1 (1.8)
Retained medullary cord	1 (1.8)
Arthrogryposis	1 (1.8)
Imperforate anus	1 (1.8)
Twin-twin transfusion syndrome	1 (1.8)
Intramedullary abscess	1 (1.8)
Human tail	1 (1.8)
Hydromyelia	1 (1.8)

Data reported as number of studies reporting (percent)

### Indications for surgery

Twenty-eight surgical indication categories were extracted from the 55 studies (Table 2), with a clear delineation between non-urologic and urologic indications. Of the non-urologic surgical indications, the five that were reported most often were nonspecific lower-extremity motor disturbances (reported in 36.4% of studies), lower-extremity/back pain (32.7%), nonspecific lower-extremity sensory disturbances (29.1%), gait abnormalities (29.1%), and nonspecific bowel dysfunction/fecal incontinence (25.5%). Urologic symptoms were also commonly reported but rarely well defined; the most common category captured was nonspecific bladder dysfunction or nonspecific urodynamic abnormalities/scores (reported by 40.0% of studies). More specific metrics included urinary incontinence/nocturesis/neurogenic bladder (23.6%), bladder capacity/volume deficiencies (23.6%), detrusor malfunction/overactivity (18.2%), bladder compliance deficiencies (16.4%), and postvoid residual volume (10.9%). Additional urodynamic metrics or urologic symptoms were captured across some studies, although none of these variables were reported in more than 10% of all studies.

**Table 2:** Indications for surgery

**Variable**	**Included studies (*n*** **= 55)**
Non-urologic symptoms	
Nonspecific lower-extremity motor deficits	20 (36.4)
Lower-extremity/back pain	18 (32.7)
Nonspecific lower-extremity sensory deficits	16 (29.1)
Gait abnormalities	16 (29.1)
Nonspecific bowel dysfunction/fecal incontinence	14 (25.5)
Nonspecific lower-extremity weakness	11 (20.0)
Orthopedic abnormalities	7 (12.7)
Spasticity	7 (12.7)
Lower-extremity muscular atrophy	6 (10.9)
Saddle anesthesia/perianal sensation changes	5 (9.1)
Hyperreflexia	3 (5.5)
Areflexia	3 (5.5)
Urologic symptoms	
Nonspecific bladder dysfunction/nonspecific urodynamics	22 (40.0)
Urinary incontinence/nocturesis/neurogenic bladder	13 (23.6)
Bladder capacity/volume deficiencies	13 (23.6)
Detrusor malfunction/overactivity	10 (18.2)
Bladder compliance deficiencies	9 (16.4)
Postvoid residual volume	6 (10.9)
Manometry/sphincter evaluation	5 (9.1)
Other urodynamics	4 (9.1)
Bladder pressure	3 (5.5)
Urinary/bladder sensory loss	3 (5.5)
History of/recurrent urinary tract infections	3 (5.5)
Voiding function	2 (3.6)
Detrusor leak point pressure	2 (3.6)
Deterioration of bladder storage	1 (1.8)
Bladder/kidney anatomic abnormalities	1 (1.8)

Data reported as number of studies reporting (percent)

### Postsurgical outcomes

After TCR, two non-urological outcomes emerged as the most commonly reported: secondary cord formation or postoperative “retethering” upon follow-up (40.0% of studies) and nonspecific motor deficits (32.7%) (Table 3). Other commonly reported non-urologic outcomes included nonspecific lower-extremity/back/perianal pain (18.2%), nonspecific gait/ambulation function (18.2%), nonspecific sensory deficits (12.7%), and nonspecific bowel function/fecal incontinence (12.7%). Among urologic outcomes, nonspecific bladder function/urinary symptoms were most often described (27.3%); bladder capacity (20.0%), bladder compliance (18.2%), urinary incontinence/enuresis/neurogenic bladder (18.2%), nonspecific urodynamics/global urodynamics score change (16.4%), and detrusor function (10.9%) were also commonly captured categories. We identified 11 additional non-urologic and 8 additional urologic parameter categories reported among ≤ 10% of studies.

**Table 3:** Postsurgical outcomes described

**Variable**	**Included studies (*n*** **= 55)**
Non-urologic outcomes	
Secondary cord formation (retethering)	22 (40.0)
Nonspecific motor deficits	18 (32.7)
Nonspecific lower-extremity/back/perianal pain	10 (18.2)
Nonspecific gait/ambulation	10 (18.2)
Nonspecific sensory deficits	7 (12.7)
Nonspecific bowel function/fecal incontinence	7 (12.7)
General symptoms/status	5 (9.1)
Anorectal manometries/sphincter function	5 (9.1)
Nonspecific lower-extremity weakness	4 (7.3)
Nonspecific neurologic deficits	3 (5.5)
Nonspecific muscular atrophy	3 (5.5)
Orthopedic abnormalities	2 (3.6)
Spasticity	2 (3.6)
Reflexes	1 (1.8)
Nonspecific lower-extremity dysfunction	1 (1.8)
Requiring orthotics	1 (1.8)
Orthopedic symptoms that required additional intervention	1 (1.8)
Urinary outcomes	
Nonspecific bladder function/urinary symptoms	15 (27.3)
Bladder capacity	11 (20.0)
Bladder compliance	10 (18.2)
Urinary incontinence/neurogenic bladder	10 (18.2)
Nonspecific urodynamics/global urodynamics score	9 (16.4)
Detrusor function	6 (10.9)
Voiding function	4 (7.3)
Leak point/capacity pressure	4 (7.3)
Post-void residual volume	4 (7.3)
Vesicoureteral reflux	2 (3.6)
Recurrent urinary tract infections	2 (3.6)
Other urodynamic measures	2 (3.6)
Video bladder neck evaluation	1 (1.8)
Urinary sensory dysfunction	1 (1.8)

Data reported as number of studies reporting (percent)

### Complications

Seven studies (12.7%) reported managing wound infections postoperatively (Table 4). Some studies also reported cerebrospinal fluid leaks (CSF) without (9.3%) and with (7.3%) surgical repair. Pseudomeningocele was described in 5.5% of reports; all other reported complication event types (11) were reported in < 5% of studies. Sixty percent of studies either reported no complications or did not disclose them.

**Table 4:** Complications as reported in each study

**Variable**	**Included studies (*n*** **= 55)**
Wound infection	7 (12.7)
CSF leak/CSF collection without repair	5 (9.1)
CSF leak requiring repair	4 (7.3)
Pseudomeningocele	3 (5.5)
Retethering	2 (3.6)
Wound dehiscence	2 (3.6)
Surgical repair of wound complications	2 (3.6)
Diminished bladder control	1 (1.8)
Ibanez type Ib complications	1 (1.8)
New neuro-orthopedic complications	1 (1.8)
Dural tear	1 (1.8)
Stitch abscess	1 (1.8)
Headache	1 (1.8)
Deep vein thrombosis	1 (1.8)
Reoperation	1 (1.8)
None/not reported	31 (56.4)

Data reported as number of studies reporting (percent)

*CSF* cerebrospinal fluid

### Spinal column-shortening study

There was only one study [68] in which spinal column shortening was used as a revision strategy after recurrent symptoms despite prior TCR. In this study, neurological outcomes were evaluated using the Japanese Orthopaedic Association (JOA) score, pain levels, visual analog scale score, urodynamics with the International Consultation on Incontinence Questionnaire-Urinary Incontinence 3 Short Form score, and bowel function with the Rintala score.

## Discussion

Here we have demonstrated the significant heterogeneity with respect to surgical indications and outcome measures in TCR for TCS. Although many studies have described surgical approaches and treatment methods, the wide disparity in outcomes reporting limits the study of this disease process. As such, meaningful meta-analyses of outcomes cannot be performed. These findings underscore the necessity for a standardized approach to outcome reporting.

To some extent, the significant heterogeneity in associated anatomical defects and surgical indications reflects the numerous etiologies of the disease. For any pathologic process with congenital and acquired causes, the formation of specific etiology-based research initiatives is necessary to better guide treatment. To date, this does not exist for TCS and represents a potential avenue for improvement. Similar strides have been made with hydrocephalus research through the concerted efforts of the Hydrocephalus Clinical Research Network [70].

### Reported surgical indications

In evaluating the various reported indications for surgical intervention, despite coalescing variables with reasonable similarity, we identified 27 different variables from 55 studies. There was a clear delineation between urologic symptoms and non-urologic symptoms, with 15 categories identified that related to urinary symptoms. Perhaps more concerning, the 5 most commonly reported surgical indications were amorphous, highly subjective, or ill-defined. These included nonspecific bladder dysfunction or nonspecific urodynamics (40.0% of studies), nonspecific lower-extremity motor disturbances (36.4%), lower-extremity/back pain (32.7%) nonspecific lower-extremity sensory disturbances (29.1%), and gait abnormalities (29.1%). Because of the general lack of objectivity in these measurements or lack of a validated scale for subjective measures, it becomes unfeasible to compare patient presentation or symptom severity among studies. This heterogeneity in patient presentation reporting leads to the significant disparity in indication categories and subsequent goals of surgery. Although specific and highly empiric data were reported in some studies, such measures were not common across the literature as a whole. For example, although objective assessment of detrusor malfunction/overactivity and bladder compliance irregularities were reported in 18.2 and 16.4% of studies, respectively, other such standardized metrics like manometry/sphincter assessment, bladder pressure, or detrusor leak point pressure were assessed in < 10% of reports.

### Reported postoperative outcomes

Although all patients included in this review underwent detethering surgery, the same issues observed in patient presentation reporting were repeated among post-TCR outcomes. After dividing outcomes into urologic and non-urologic categories, we identified 31 sufficiently distinct postoperative outcome categories. The most commonly reported outcome was sufficiently objective (cord retethering rate), but it was followed in frequency by nondescript and nonspecific motor/neuromotor (32.7%) and bladder (27.3%) deficits and symptoms. In total, of the 31 reported outcome categories (both non-urologic and urologic), we characterized 12 as being nonspecific or generalized. The most empiric postsurgical outcomes were among the urologic measures, as bladder capacity and compliance were evaluated in 20.0 and 18.2% of studies, respectively. Other objective urinary measures were not commonly available. We often observed reports describing “urodynamics” as a primary outcome, but it became clear that the urodynamic measurements performed were inconsistent from study to study. For example, the detailed urodynamic outcomes captured by Alzahrani et al. [18] included total cystometric bladder capacity (TCBC), intravesical pressure at TCBC, detrusor leak point pressure, and compliance at TCBC, as well as 75% bladder capacity, uninhibited bladder contractions, detrusor sphincter dyssynergia, and percentage change in bladder capacity before and after TCR. Lavallée et al. [36] reported both similar and different urodynamic measurements by describing mean bladder capacity and mean compliance, the number of detrusor contractions during bladder filling, and patient bladder capacity/compliance in relation to age group.

The limited commonality among TCS studies is evident in the meta-analyses that have been published to date. For example, in their recently published systematic review and meta-analysis regarding minimally invasive surgery for pediatric occult TCS, Xu et al. [71] were able to include only six studies, with the only consistently reported outcome measure being the postsurgical nonimprovement rate. Notably, they were forced to exclude six eligible studies because of insufficient data for observational indicators regarding nonimprovement description. Similarly, in their 2020 systematic review and meta-analysis of surgical detethering in adult TCS, O’Conner et al. [72] reported nondescript presentation symptoms, including pain, motor deficits, sensory deficits, bladder dysfunction, and bowel dysfunction. With 97% of the cohort (708/730 patients) undergoing TCR, this study was limited to only reporting patient outcomes as either improved, unchanged, or worsened compared with their presenting symptomatology (i.e., motor, sensory, bladder, or bowel deficits/dysfunction or pain). Quantitative metrics that detailed the degree of improvement in symptomatology were not described. McVeigh et al. [13] likewise completed a systematic review and meta-analysis of spinal column shortening as a management strategy for TCS. Describing 15 studies and 191 patients, postoperative outcomes were reported as nonquantified improvement in patient pain, weakness, and bladder/bowel dysfunction. These meta-analyses make it clear that the collation of individual studies and the derivation of high-level conclusions and management technique comparisons are greatly hampered by the current heterogeneous state of TCR outcomes reporting.

### Classification systems in neurosurgery

Across neurosurgery, the management of complex or frequent pathologies has been simplified by the formation of validated classification systems or grading scales. Among the numerous examples are the NIH Stroke Scale [73], the Glasgow Coma Scale in neurotrauma [74], the Hunt and Hess Scale to predict mortality in subarachnoid hemorrhage [75], the American Spinal Injury Association Impairment Scale in spinal cord injury [77–84]. Each of these systems has streamlined prognostication and outcome prediction within its respective scope. Furthermore, these systems facilitate efficient patient comparison for higher-level analysis among institutions and published outcomes data, which is vital as the U.S. moves toward a value-based healthcare economy.

### Toward future TCS classification formation and registry formation

It is clear from our analysis that TCS management suffers from the lack of a common outcome reporting tool or instrument. Although the reports we assessed often contained vague and unspecified patient symptoms and outcomes, it is apparent that several domains should be accounted for in a future TCS grading system. These would potentially include both subjective and objective measures, such as lower-extemity motor and sensory components, lower-extremity or back pain assessments, and quantifiable urodynamic parameters such as bladder capacity, bladder compliance, and post-void residual volume [85]. Likewise, in the absence of a grading system, future work would benefit from the creation of a prospective TCS registry with common data elements. Such a registry would allow for the study of important questions, such as the comparative effectiveness of minimally invasive vs. open surgical treatment and the use of spinal column shortening vs. traditional TCR. Etiology-based research initiatives will also profit from such an endeavor.

### Limitations

Our study is subject to the limitations common to systematic reviews including imperfect catchment strategy or search term design. Additionally, our synthesis of reported variables into common umbrella categories introduces opportunities for mischaracterization. Despite these potential limitations, we believe that the present work illustrates the challenging task of evaluating TCR outcomes for TCS across currently available literature and highlights potential avenues for future improvement.

## Conclusions

TCS surgical literature is highly varied in the reporting of patient presentation, surgical indications, and surgical outcomes. The lack of a validated patient-reported outcome measurement and common and consistent objective measures inhibits higher-level analysis. The development and validation of a standardized scale or classification system would significantly benefit future TCS research and surgical management.

## Data Availability

Data generated or analyzed during the study are available from the corresponding author by request.

## Declarations

### Copyright statement

“I am a military service member. This work was prepared as part of my official duties. Title 17, U.S.C., §105 provides that copyright protection under this title is not available for any work of the U.S. Government. Title 17, U.S.C., §101 defines a U.S. Government work as a work prepared by a military service member or employee of the U.S. Government as part of that person’s official duties.”

## References

[1] Yamada S, Iacono RP, Andrade T, Mandybur G, Yamada BS (1995). Pathophysiology of tethered cord syndrome. Neurosurg Clin N Am.

[2] Yamada S, Won DJ, Yamada SM (2004). Pathophysiology of tethered cord syndrome: correlation with symptomatology. Neurosurg Focus.

[3] Yamada S, Zinke DE, Sanders D (1981). Pathophysiology of "tethered cord syndrome". J Neurosurg.

[4] Agarwalla PK, Dunn IF, Scott RM, Smith ER (2007). Tethered cord syndrome. Neurosurg Clin N Am.

[5] Barutçuoğlu M, Selçuki M, Umur AS, Mete M, Gurgen SG, Selcuki D (2016). Scoliosis may be the first symptom of the tethered spinal cord. Indian J Orthop.

[6] Jackson T, Jones A, Miller N, Georgopoulos G (2019). Clubfoot and tethered cord syndrome: results of treatment with the Ponseti Method. J Pediatr Orthop.

[7] Lew SM, Kothbauer KF (2007). Tethered cord syndrome: an updated review. Pediatr Neurosurg.

[8] Shukla M, Sardhara J, Sahu RN, Sharma P, Behari S, Jaiswal AK, Srivastava AK, Mehrotra A, Das KK, Bhaisora KS (2018). Adult versus pediatric tethered cord syndrome: clinicoradiological differences and its management. Asian J Neurosurg.

[9] Bhimani AD, Selner AN, Patel JB, Hobbs JG, Esfahani DR, Behbahani M, Zayyad Z, Nikas D, Mehta AI (2019). Pediatric tethered cord release: an epidemiological and postoperative complication analysis. Journal of Spine Surgery.

[10] Solmaz I, Izci Y, Albayrak B, Cetinalp E, Kural C, Sengul G, Gocmez C, Pusat S, Tuzun Y (2011). Tethered cord syndrome in childhood: special emphasis on the surgical technique and review of the literature with our experience. Turk Neurosurg.

[11] Hoffman HJ, Hendrick EB, Humphreys RP (1976). The tethered spinal cord: its protean manifestations, diagnosis and surgical correction. Childs Brain.

[12] Catmull S, Ashurst J (2019). Tethered cord syndrome. Clin Pract Cases Emerg Med.

[13] McVeigh LG, Anokwute MC, Chen S, Jea A (2022). Spinal column shortening for tethered cord syndrome: a systematic review and individual patient data meta-analysis. J Neurosurg Pediatr.

[14] Stewart LA, Clarke M, Rovers M, Riley RD, Simmonds M, Stewart G, Tierney JF (2015). Preferred reporting items for systematic review and meta-analyses of individual participant data: the PRISMA-IPD Statement. JAMA.

[15] Ouzzani M, Hammady H, Fedorowicz Z, Elmagarmid A (2016). Rayyan — a web and mobile app for systematic reviews. Syst Rev.

[16] Abrahamsson K, Olsson I, Sillén U (2007) Urodynamic findings in children with myelomeningocele after untethering of the spinal cord. J Urol 177: 331–334. discussion 334 10.1016/j.juro.2006.08.146 17162083

[17] Altiok H, Riordan A, Graf A, Krzak J, Hassani S (2016). Response of scoliosis in children with myelomeningocele to surgical release of tethered spinal cord. Top Spinal Cord Inj Rehabil.

[18] Alzahrani A, Alsowayan O, Farmer JP, Capolicchio JP, Jednak R, El-Sherbiny M (2016). Comprehensive analysis of the clinical and urodynamic outcomes of secondary tethered spinal cord before and after spinal cord untethering. J Pediatr Urol.

[19] Balkan E, Kiliç N, Avşar I, Boyaci S, Aksoy K, Doğruyol H (2001). Urodynamic findings in the tethered spinal cord: the effect of tethered cord division on lower urinary tract functions. Eur J Pediatr Surg.

[20] Beaumont A, Muszynski CA, Kaufman BA (2007). Clinical significance of terminal syringomyelia in association with pediatric tethered cord syndrome. Pediatr Neurosurg.

[21] Behbahani M, Shlobin N, Rosen C, Yerkes E, Swaroop V, Lam S, Bowman R (2020). Multidisciplinary management of tethered spinal cord syndrome in children: operationalizing an outpatient patient-centered workflow. J Multidiscip Healthc.

[22] Broderick KM, Munoz O, Herndon CD, Joseph DB, Kitchens DM (2015). Utility of urodynamics in the management of asymptomatic tethered cord in children. World J Urol.

[23] Cha S, Wang KC, Park K, Shin HI, Lee JY, Chong S, Kim K (2018). Predictive value of intraoperative bulbocavernosus reflex during untethering surgery for post-operative voiding function. Clin Neurophysiol.

[24] Chern JJ, Dauser RC, Whitehead WE, Curry DJ, Luerssen TG, Jea A (2011) The effect of tethered cord release on coronal spinal balance in tight filum terminale. Spine (Phila Pa 1976) 36: E944–949 10.1097/BRS.0b013e3181fc2edd 21289577

[25] Cornette L, Verpoorten C, Lagae L, Van Calenbergh F, Plets C, Vereecken R, Casaer P (1998). Tethered cord syndrome in occult spinal dysraphism: timing and outcome of surgical release. Neurology.

[26] Destro F, Canazza L, Meroni M, Selvaggio G, Parazzini C, Valentini L, Riccipetitoni G (2018). Tethered cord and anorectal malformations: a case series. Eur J Pediatr Surg.

[27] Edström E, Wesslén C, Fletcher-Sandersjöö A, Elmi-Terander A, Sandvik U (2022). Filum terminale transection in pediatric tethered cord syndrome: a single center, population-based, cohort study of 95 cases. Acta Neurochir (Wien).

[28] Erkan K, Unal F, Kiris T, Karalar T (2000). Treatment of terminal syringomyelia in association with tethered cord syndrome: clinical outcomes with and without syrinx drainage. Neurosurg Focus.

[29] Geyik M, Geyik S, Şen H, Pusat S, Alptekin M, Yılmaz AE, Nazik M, Erkutlu İ (2016). Urodynamic outcomes of detethering in children: experience with 46 pediatric patients. Childs Nerv Syst.

[30] Glenn C, Cheema AA, Safavi-Abbasi S, Gross NL, Martin MD, Mapstone TB (2015). Spinal cord detethering in children with tethered cord syndrome and Chiari type 1 malformations. J Clin Neurosci.

[31] Guerra LA, Pike J, Milks J, Barrowman N, Leonard M (2006). Outcome in patients who underwent tethered cord release for occult spinal dysraphism. J Urol.

[32] Hayashi T, Kimiwada T, Kohama M, Shirane R, Tominaga T (2018). Minimally invasive surgical approach to filum lipoma. Neurol Med Chir (Tokyo).

[33] Hayashi T, Kimiwada T, Shirane R, Tominaga T (2022). Retethering risk in pediatric spinal lipoma of the conus medullaris. J Neurosurg Pediatr.

[34] Inoue M, Uchida K, Otake K, Nagano Y, Shimura T, Hashimoto K, Matsushita K, Koike Y, Matsubara T, Kusunoki M (2017). Long-term functional outcome after untethering surgery for a tethered spinal cord in patients with anorectal malformations. Pediatr Surg Int.

[35] Lagae L, Verpoorten C, Casaer P, Vereecken R, Fabry G, Plets C (1990). Conservative versus neurosurgical treatment of tethered cord patients. Z Kinderchir.

[36] Lavallée LT, Leonard MP, Dubois C, Guerra LA (2013). Urodynamic testing—is it a useful tool in the management of children with cutaneous stigmata of occult spinal dysraphism?. J Urol.

[37] Lee JY, Phi JH, Cheon JE, Kim SK, Kim IO, Cho BK, Wang KC (2012). Preuntethering and postuntethering courses of syringomyelia associated with tethered spinal cord. Neurosurgery.

[38] Lee SB, Im YJ, Jung JH, Do MT, Lee JY, Wang KC, Park K (2022). Clinical and urodynamic features of secondary tethered cord syndrome: how can they be found longitudinally?. Neurourol Urodyn.

[39] McGirt MJ, Mehta V, Garces-Ambrossi G, Gottfried O, Solakoglu C, Gokaslan ZL, Samdani A, Jallo GI (2009). Pediatric tethered cord syndrome: response of scoliosis to untethering procedures. J Neurosurg Pediatr.

[40] McLone DG, Herman JM, Gabrieli AP, Dias L (1990). Tethered cord as a cause of scoliosis in children with a myelomeningocele. Pediatr Neurosurg.

[41] Mehta VA, Gottfried ON, McGirt MJ, Gokaslan ZL, Ahn ES, Jallo GI (2011). Safety and efficacy of concurrent pediatric spinal cord untethering and deformity correction. J Spinal Disord Tech.

[42] Metcalfe PD, Luerssen TG, King SJ, Kaefer M, Meldrum KK, Cain MP, Rink RC, Casale AJ (2006) Treatment of the occult tethered spinal cord for neuropathic bladder: results of sectioning the filum terminale. J Urol 176: 1826–1829. discussion 1830 10.1016/j.juro.2006.04.090 16945660

[43] Meyrat BJ, Vernet O, Berger D, de Tribolet N (1993). Pre- and postoperative urodynamic and anorectal manometric findings in children operated upon for a primary tethered cord. Eur J Pediatr Surg.

[44] Mualem W, Nathani KR, Durrani S, Zamanian C, Ghaith AK, Michalopoulos GD, Rotter J, Daniels D, Bydon M (2023). Utilizing pre- and postoperative radiological parameters to predict surgical outcomes following untethering for tethered cord syndrome in a pediatric population. J Neurosurg Pediatr.

[45] Nogueira M, Greenfield SP, Wan J, Santana A, Li V (2004) Tethered cord in children: a clinical classification with urodynamic correlation. J Urol 172:1677–1680. discussion 1680 10.1097/01.ju.0000140140.75441.f0 15371788

[46] Ogiwara H, Joko M, Takado M, Uematsu K, Kameda M, Sasaki N, Kitagawa M, Morota N (2015). Duration of the horizontal decubitus position for prevention of cerebrospinal fluid leakage following transection of a tight filum terminale. J Neurosurg Pediatr.

[47] Ostling LR, Bierbrauer KS, Ct K (2012). Outcome, reoperation, and complications in 99 consecutive children operated for tight or fatty filum. World Neurosurg.

[48] Sadrameli SS, Chu JK, Chan TM, Steele WJ, Curry DJ, Lam SK (2019). Minimally invasive tubular tethered cord release in the pediatric population. World Neurosurg.

[49] Samuels R, McGirt MJ, Attenello FJ, Garcés Ambrossi GL, Singh N, Solakoglu C, Weingart JD, Carson BS, Jallo GI (2009). Incidence of symptomatic retethering after surgical management of pediatric tethered cord syndrome with or without duraplasty. Childs Nerv Syst.

[50] Schoenmakers MA, Gooskens RH, Gulmans VA, Hanlo PW, Vandertop WP, Uiterwaal CS, Helders PJ (2003). Long-term outcome of neurosurgical untethering on neurosegmental motor and ambulation levels. Dev Med Child Neurol.

[51] Seki T, Hida K, Yano S, Sasamori T, Hamauch S, Koyanagi I, Houkin K (2016). Surgical outcome of children and adolescents with tethered cord syndrome. Asian Spine J.

[52] Seki T, Hida K, Yano S, Houkin K (2018). Surgical outcomes of pediatric patients with asymptomatic tethered cord syndrome. Asian Spine J.

[53] Selden NR, Nixon RR, Skoog SR, Lashley DB (2006). Minimal tethered cord syndrome associated with thickening of the terminal filum. J Neurosurg.

[54] Shahjouei S, Hanaei S, Habibi Z, Hoseini M, Ansari S, Nejat F (2016). Randomized clinical trial of acetazolamide administration and/or prone positioning in mitigating wound complications following untethering surgeries. J Neurosurg Pediatr.

[55] Sim J, Shim Y, Kim KH, Kim SK, Lee JY (2021). Features of the filum terminale in tethered cord syndrome with focus on pathology. J Korean Neurosurg Soc.

[56] Stavrinou P, Kunz M, Lehner M, Heger A, Müller-Felber W, Tonn JC, Peraud A (2011). Children with tethered cord syndrome of different etiology benefit from microsurgery-a single institution experience. Childs Nerv Syst.

[57] Steinbok P, MacNeily AE, Hengel AR, Afshar K, Landgraf JM, Hader W, Pugh J (2016). Filum section for urinary incontinence in children with occult tethered cord syndrome: a randomized, controlled pilot study. J Urol.

[58] Thuy M, Chaseling R, Fowler A (2015). Spinal cord detethering procedures in children: a 5 year retrospective cohort study of the early post-operative course. J Clin Neurosci.

[59] Tuuha SE, Aziz D, Drake J, Wales P, Kim PC (2004). Is surgery necessary for asymptomatic tethered cord in anorectal malformation patients?. J Pediatr Surg.

[60] Uchida K, Inoue M, Matsubara T, Otake K, Koike Y, Okugawa Y, Kawamoto A, Miki C, Kusunoki M (2007). Evaluation and treatment for spinal cord tethering in patients with anorectal malformations. Eur J Pediatr Surg.

[61] Udayakumaran S, Rathod CT (2018). Tailored strategies to manage cerebrospinal fluid leaks or pseudomeningocele after surgery for tethered cord syndrome. World Neurosurg.

[62] Udayakumaran S, Karthika KS, Nair NS, George M, Gopinath S (2021) Prognostication of the neurological outcome of tethered cord based on intraoperative neuromonitoring findings: how close can we get? Br J Neurosurg. online ahead of print 10.1080/02688697.2021.1940855 34459322

[63] Valentini LG, Babini M, Cordella R, Beretta E, Destro F, Murabito P, Caldiroli D, Devigili G, Selvaggio G (2021). Early de-tethering: analysis of urological and clinical consequences in a series of 40 children. Childs Nerv Syst.

[64] Vepakomma D, Kumar N, Alladi A (2019). Tethered cord syndrome—role of early surgery. J Indian Assoc Pediatr Surg.

[65] Yener S, Thomas DT, Hicdonmez T, Dagcinar A, Bayri Y, Kaynak A, Dagli TE, Tugtepe H (2015). The effect of untethering on urologic symptoms and urodynamic parameters in children with primary tethered cord syndrome. Urology.

[66] Meyrat BJ, Tercier S, Lutz N, Rilliet B, Forcada-Guex M, Vernet O (2003). Introduction of a urodynamic score to detect pre- and postoperative neurological deficits in children with a primary tethered cord. Childs Nerv Syst.

[67] Robbins JW, Lundy PA, Gard AP, Puccioni MJ (2015). Perineal pain secondary to tethered cord syndrome: retrospective review of single institution experience. Childs Nerv Syst.

[68] Wang H, Xu T, Sun J, Wang Y, Sun K, Xu X, Zhang B, Guo Y, Shi J (2019). Homogeneous spinal-shortening axial decompression as a revision surgery after untethering surgery in pediatric patients with tethered cord syndrome. World Neurosurg.

[69] Bowman RM, Mohan A, Ito J, Seibly JM, McLone DG (2009). Tethered cord release: a long-term study in 114 patients. J Neurosurg Pediatr.

[70] Kestle JRW, Riva-Cambrin J (2019). Prospective multicenter studies in pediatric hydrocephalus. J Neurosurg Pediatr.

[71] Xu K, He J, Wang L (2022). A systematic review and meta-analysis of minimally invasive surgery in children with occult tethered cord syndrome. Transl Pediatr.

[72] O'Connor KP, Smitherman AD, Milton CK, Palejwala AH, Lu VM, Johnston SE, Homburg H, Zhao D, Martin MD (2020). Surgical treatment of tethered cord syndrome in adults: a systematic review and meta-analysis. World Neurosurgery.

[73] Lyden P, Brott T, Tilley B, Welch KM, Mascha EJ, Levine S, Haley EC, Grotta J, Marler J (1994). Improved reliability of the NIH Stroke Scale using video training. NINDS TPA Stroke Study Group Stroke.

[74] Jain S, Iverson LM (2023). Glasgow Coma Scale.

[75] Hunt W, Hess R (1968). Surgical risk as related to time of intervention in the repair of intracranial aneurysms. J Neurosurg.

[76] Roberts TT, Leonard GR, Cepela DJ (2017). Classifications in brief: American Spinal Injury Association (ASIA) Impairment Scale. Clin Orth Relat Res.

[77] Kutty S (2022). A summary of common grading systems used in neurosurgical practice. Surg Neurol Int.

[78] Biffl WL, Moore EE, Offner PJ, Brega KE, Franciose RJ, Burch JM (1999). Blunt carotid arterial injuries: implications of a new grading scale. J Trauma Acute Care Surg.

[79] Fang Y, Pei Z, Chen H, Wang R, Feng M, Wei L, Li J, Zhang H, Wang S (2021). Diagnostic value of Knosp grade and modified Knosp grade for cavernous sinus invasion in pituitary adenomas: a systematic review and meta-analysis. Pituitary.

[80] Fisher CM, Kistler JP, Davis JM (1980). Relation of cerebral vasospasm to subarachnoid hemorrhage visualized by computerized tomographic scanning. Neurosurgery.

[81] Spetzler RF, Martin NA (1986). A proposed grading system for arteriovenous malformations. J Neurosurg.

[82] Kulkarni AV, Drake JM, Mallucci CL, Sgouros S, Roth J, Constantini S (2009). Endoscopic third ventriculostomy in the treatment of childhood hydrocephalus. J Pediatr.

[83] House JW, Brackmann DE (1985). Facial nerve grading system. Otolaryngol Head Neck Surg.

[84] Quinn TJ, Dawson J, Walters M (2008). Dr John Rankin; his life, legacy and the 50th anniversary of the Rankin Stroke Scale. Scott Med J.

[85] Yao M, Simoes A (2023). Urodynamic testing and interpretation.

